# High resolution proteomics of *Aedes aegypti* salivary glands infected with either dengue, Zika or chikungunya viruses identify new virus specific and broad antiviral factors

**DOI:** 10.1038/s41598-021-03211-0

**Published:** 2021-12-08

**Authors:** Avisha Chowdhury, Cassandra M. Modahl, Dorothée Missé, R. Manjunatha Kini, Julien Pompon

**Affiliations:** 1 Department of Biological Science, National University of Singapore, Singapore, Singapore; 2 MIVEGEC, Univ. Montpellier, IRD, CNRS, Montpellier, France; 3 Department of Pharmacology, Yong Loo Lin School of Medicine, National University of Singapore, Singapore, Singapore; 4 Present Address: Programme in Emerging Infectious Diseases, Duke-NUS Medical School, Singapore, Singapore; 5 Present Address: Liverpool School of Tropical Medicine, Pembroke Place, Liverpool, UK; 6 Present Address: MIVEGEC, Univ. Montpellier, IRD, CNRS, Montpellier, France

**Keywords:** Dengue virus, Entomology

## Abstract

Arboviruses such as dengue (DENV), Zika (ZIKV) and chikungunya (CHIKV) viruses infect close to half a billion people per year, and are primarily transmitted through *Aedes aegypti* bites. Infection-induced changes in mosquito salivary glands (SG) influence transmission by inducing antiviral immunity, which restricts virus replication in the vector, and by altering saliva composition, which influences skin infection. Here, we profiled SG proteome responses to DENV serotype 2 (DENV2), ZIKV and CHIKV infections by using high-resolution isobaric-tagged quantitative proteomics. We identified 218 proteins with putative functions in immunity, blood-feeding or related to the cellular machinery. We observed that 58, 27 and 29 proteins were regulated by DENV2, ZIKV and CHIKV infections, respectively. While the regulation patterns were mostly virus-specific, we separately depleted four uncharacterized proteins that were upregulated by all three viral infections to determine their effects on these viral infections. Our study suggests that gamma-interferon responsive lysosomal thiol-like (GILT-like) has an anti-ZIKV effect, adenosine deaminase (ADA) has an anti-CHIKV effect, salivary gland surface protein 1 (SGS1) has a pro-ZIKV effect and salivary gland broad-spectrum antiviral protein (SGBAP) has an antiviral effect against all three viruses. The comprehensive description of SG responses to three global pathogenic viruses and the identification of new restriction factors improves our understanding of the molecular mechanisms influencing transmission.

## Introduction

Arboviruses like dengue (DENV), Zika (ZIKV) and chikungunya (CHIKV) viruses are primarily spread through the bites of *Aedes aegypti* mosquitoes^1^. DENV and ZIKV belong to the *Flavivirus* genus (Flaviviridae family), while CHIKV is an *Alphavirus* (Togaviridae family). These viruses infect more than 400 million people yearly, mostly in tropical and sub-tropical countries where the environment is conducive to natural mosquito breeding^2^. In the absence of effective vaccines and drugs^3^, mitigation of these mosquito-borne diseases relies on vector population control. Insecticide-based controls often fail to curb outbreaks partly due to insecticide resistance^4,5^. Alternatively, novel biological strategies are being developed to limit vector populations. Population suppression or replacement by releasing *Wolbachia*-infected mosquitoes is currently being evaluated in field trials in several countries^6^. However, the ability to scale up the suppression strategy and the sustainability of the replacement strategy ^7^ remain to be validated. The production of pathogen-refractory mosquitoes by genetic modification offers another promising vector-targeted strategy^8,8^. In this regard, identification of pro- and anti-viral factors in mosquitoes is a prerequisite.

While biting an infected host, female mosquitoes ingest viruses that first infect the mosquito midgut and then disseminate throughout the mosquito body before finally reaching the salivary glands (SG), from where the virus is secreted during a subsequent bite. The time the virus takes from entering the mosquito midgut until it is secreted in the mosquito saliva is termed the extrinsic incubation period (EIP). EIP varies among virus species. EIP for flaviviruses like DENV and ZIKV is estimated to be between 10 and 14 days^9,10^ and for alphaviruses like CHIKV between two to nine days^11^. *Aedes aegypti* possesses pathogen-responsive innate immune pathways in midgut, hemolymph and SG, which limit virus infection, and thus transmission^12,13^. Transcriptomics studies of SG showed that the induction of Toll and IMD pathways by DENV serotype 2 (DENV2) leads to the production of a cecropin-like antimicrobial peptide with anti-DENV2, anti-CHIKV and anti-*Leishmania* properties^14^. Using high-throughput RNA sequencing, we recently showed that DENV2, ZIKV and CHIKV infections trigger a broad antiviral response through the c-jun N terminal kinase (JNK) pathway, which activates complement and apoptotic effectors^15^. Alternatively, other factors unrelated to the canonical immune pathways have been implicated in the regulation of DENV2 infection in SG^16^. While multiple evidence indicate that mosquito SG response modulates infection, additional studies are required to characterize the response at the proteomic level, identify novel viral factors and determine how infection-regulated proteins influence viral infection.

SG infection also alters the composition of saliva, thereby influencing blood acquisition and skin infection^17–19^. Mosquito saliva contains a cocktail of biologically active molecules with functions in hemostasis, inflammation and immunity^20,21^. Among others, *A. aegypti* saliva contains a vasodilatory tachykinin decapeptide named sialokinin, a factor Xa-directed anticoagulant and an anti-platelet apyrase, all of which may facilitate blood acquisition by preventing clotting to maintain steady blood flow^22–24^. Immune-modulators such as a secreted 387 kDa protein can suppress cytokine release and proliferation of T and B cells in mouse splenocytes in vitro^25^. *Aedes aegypti* salivary gland extract (SGE) induces apoptosis of lymphocytes (CD4^+^ and CD8^+^ T cells, and B cells) in a caspase-3 and caspase-8 dependant pathway^26^. A venom allergen-1 protein detected in *A. aegypti* saliva was recently found to enhance DENV2 and ZIKV infection in skin cells by augmenting autophagy^27^. Alternatively, salivary proteins can also inhibit skin infection. A 30 kDa collagen-binding protein called aegyptin^28^ and a D7 protein^29^ reduce DENV2 multiplication. Characterization of SG proteomic response to infection will inform about changes in saliva composition, which can affect transmission.

Previous studies of the *A. aegypti* SG proteome used uninfected mosquitoes and one- or two-dimensional gel electrophoresis (DGE) coupled with mass spectrometry (MS) to identify a few proteins^30,31^. Recently, using high-resolution MS, 1,208 proteins were detected in uninfected *A. aegypti* SG^32^, although these included proteins identified by only one unique peptide. To our knowledge, only two studies for DENV2, one for CHIKV and none for ZIKV reported SG proteomic response to infection with low resolution MS^33–35^. Here, to bridge this knowledge gap in SG proteomic response to viral infections, we deployed high-resolution MS with isobaric tag for relative and absolute quantitation (iTRAQ) on DENV2-, ZIKV- and CHIKV-infected and non-infected *A. aegypti* SG. We identified 218 proteins using a custom protein database and described those with functions related to immunity, blood feeding, digestion, metabolism and ribosome, stress and mitochondria. DENV2 infection regulated the expression of 58 proteins, ZIKV infection regulated the expression of 27 proteins, and CHIKV infection regulated the expression of 29 proteins. The majority of differentially expressed proteins (DEP) were specific to infection with one virus, however we identified four proteins that changed in abundance in response to infection with all four viruses examined. We then determined the effects of knockdown of these four proteins in SG viral infection. We provide evidence for an anti-ZIKV function of gamma-interferon responsive lysosomal thiol-like (GILT-like), an anti-CHIKV function of an adenosine deaminase (ADA), a pro-ZIKV function of salivary gland surface protein 1 (SGS1) and an antiviral function against all three viruses of a protein we named salivary gland broad-spectrum antiviral protein (SGBAP).

## Methods

### Ethics declaration

The protocols were conducted in accordance with the relevant guidelines and regulations.

### Mosquito rearing

*Aedes aegypti* mosquitoes were collected in Singapore in 2010, and reared in an insectary thereafter. Eggs were hatched in MilliQ water. Larvae were kept at a density of 2.5–3 larvae/cm^2^ in shallow water and fed on a mixture of TetraMin fish flakes (Tetra, Germany) and yeast and liver powder (MP Biomedicals, France) at a 1:2 ratio. Adults were maintained in a 30 × 30 × 30 cm cage with 10% sucrose solution (1st Base, Singapore) ad libitum. Mosquitoes were maintained at 28 °C and 50% relative humidity with a 12 h:12 h light: dark cycle.

### Viruses

Dengue virus serotype 2 PVP110 was isolated from a patient enrolled in the Early DENgue infection and outcome study (EDEN) conducted in Singapore in 2008^36^. Zika virus Paraiba_01/2015 was isolated from a febrile female in the state of Paraiba, Brazil in 2015^37^. Chikungunya virus SGP011 was isolated from a patient at the National University Hospital in Singapore in 2008^38^. DENV2 and ZIKV isolates were propagated in C6/36 (CRL-1660) and CHIKV in Vero (CCL-81) cell lines. Virus stocks were titered using a BHK-21 cell virus plaque assay as previously described^39^, aliquoted and stored at − 80 °C until use.

### Mosquito infection

Three- to five-day old female mosquitoes were starved for 24 h and fed on an infectious blood meal containing 40% volume of washed erythrocytes from specific pathogen free (SPF) pig’s blood (Prestige BioResearch, Singapore), 5% 10 mM ATP (Sigma-Aldrich, USA), 5% human serum (Corning human AB serum, USA) and 50% virus solution in RPMI media (Gibco, USA), using Hemotek membrane feeder system (Discovery Workshops, UK). The virus titers in blood meals were 2 × 10^7^ pfu/ml for DENV2, 6 × 10^6^ pfu/ml for ZIKV, and 1.5 × 10^8^ pfu/ml for CHIKV, which all resulted in 100% SG infection^15^. Bloodmeal titers were validated by plaque assay using BHK-21 cells. Control mosquitoes were fed with the same blood meal composition except for the virus solution, which was replaced by RPMI media. Following oral feeding, fully engorged females were selected and kept in a cage with ad libitum access to a 10% sucrose solution in an incubation chamber with conditions similar to insect rearing.

For inoculation, mosquitoes were cold-anesthetized and intrathoracically injected with 0.5 pfu of either DENV2, ZIKV or CHIKV using a Nanoject-II (Drummond scientific company, USA). The same volume of RPMI media was injected as control. Virus inoculation was conducted four days post dsRNA injection.

### Sample preparation and iTRAQ labeling

Mosquito SG were dissected and collected in 1X phosphate buffer saline (pH 7.4, Cytiva HyClone, USA) at 14 days post oral infection (dpi) for DENV2 and ZIKV, and seven dpi for CHIKV. Ninety pairs of SG were pooled together for each condition, and freeze-thawed twice. The samples were finally homogenized at room temperature (RT) for 40 s using mini beadbeater-96 (Biospec Products, USA) and centrifuged to collect the supernatant as SGE. The protein content of each sample was normalized to 100 µg based on their concentration as measured by Pierce BCA protein assay kit (Thermo Fisher Scientific, USA). SGE were denatured, alkylated, trypsin (Promega) digested, and labeled using iTRAQ 8plex Protein quantitation kit (AB SCIEX, Singapore) following the manufacturer’s protocol. Each condition (i.e., DENV, ZIKV or CHIKV infection and age-matched controls) was conducted in triplicate.

### LC–MS/MS analysis

The 1st dimension of peptide separation was conducted using an Eksigent nanoLC Ultra and ChiPLC-nanoflex (USA) in TrapElute configuration. Subsequently, the samples were loaded on a 200 μm × 0.5 mm column and eluted on an analytical 75 μm × 15 cm column (ChromXP C18-CL, 3 μm). A gradient formed by mobile phase A (2% acetonitrile, 0.1% formic acid) and mobile phase B (98% acetonitrile, 0.1% formic acid) was used to separate 2 and 5 μl of the sample at a 0.3 μl/min flow rate. The following gradient elution was used for peptide separation: 0 to 5% of mobile phase B in 1 min, 5 to 12% of mobile phase B in 15 min, 12 to 30% of mobile phase B in 114 min, 30 to 90% of mobile phase B in 2 min, 90% for 7 min, 90 to 5% in 3 min and finally held at 5% of mobile phase B for 13 min. The tandem MS analysis was performed using a 5600 TripleTOF system (AB SCIEX, USA) under Information Dependent Acquisition (IDA) mode. The mass range of 400–1800 m/z and accumulation times of 250 millisec per spectrum were chosen for precursor ion selection. MS/MS analysis was performed on the 20 most abundant precursors (accumulation time: 100 millisec) per cycle with 15 s dynamic exclusion. Recording of MS/MS was acquired under high sensitivity mode with rolling collision energy and adjusted capillary electrophoresis (CE) when iTRAQ reagent use was selected.

### Protein identification and quantification

Peptide identification and quantification was carried out on the ProteinPilot 5.0 software Revision 4769 (AB SCIEX, Singapore) using the Paragon database search algorithm (5.0.0.0.4767) and the integrated false discovery rate (FDR) analysis function. Spectra were searched against a custom protein database including *Aedes* VectorBase (VB) (AAegL3.3 on July 2017 and AAegL5.1 on February 2019), *Aedes* UniProt (February 2019), and a translated de novo assembled *A. aegypti* SG transcriptome (unpublished) based on our previous work^15^. Within this database, we also included DENV2, ZIKV and CHIKV proteins downloaded from the National Center for Biotechnology Information (accessed July 2017). For peptide identification the tolerance for MS was set at 0.05 Da and for MS/MS was kept at 0.1 Da. The MS/MS spectra obtained were searched using the following user-defined search parameters: Sample Type: iTRAQ 8-plex (Peptide Labeled); Cysteine Alkylation: methyl methanethiosulfonate; Digestion: Trypsin; Instrument: TripleTOF5600; Special Factors: None; Species: None; ID Focus: Biological Modification; Database for *Aedes* VB Search Effort: Thorough; and FDR Analysis: Yes. The MS/MS spectra were searched against a decoy database to estimate the FDR for peptide identification. The decoy database consisted of reversed protein sequences from the same custom protein database as mentioned earlier. Different modification states of the same peptide sequences were considered distinct by the software. Peptides with confidence scores ≥ 95% were considered identified, and proteins with at least two unique identified peptides were quantified. Proteins were identified as upregulated when they had an iTRAQ ratio above 1.5 (p-value < 0.05) and downregulated when they had the ratio below 0.67 (p-value < 0.05). Proteins with a ratio from 0.67 to 1.5 were considered not regulated. Functional annotations were assigned using Blast2go software using the *Aedes* database from VectorBase (AaegL3.3), and the Diamond algorithm used to search all FlyBase proteins to identify *Drosophila melanogaster* homologs, from which we extrapolated the potential function for *A. aegypti* proteins^40,41^. Raw iTRAQ data has been submitted to the mass spectrometry interactive virtual environment (MassIVE) with the accession code MSV000087564.

### Phylogenetic analysis of SGBAP in different mosquito species

A phylogenetic tree for SGBAP and its homologs was inferred by using the maximum likelihood method and general time reversible model^42^. cDNA sequences from twenty-one homologs in *Aedes aegypti*, *Aedes albopictus* and *Culex quinquefasciatus* were identified from paralogs and orthologs in VectorBase. A bootstrap consensus tree inferred from 1000 replicates and was taken to represent the evolutionary history of the taxa analyzed. Trees for heuristic search were obtained by applying the Neighbor-Join and BioNJ algorithms to a matrix of pairwise distances estimated using the maximum composite likelihood (MCL) approach and then selecting the topology with superior log likelihood value. A discrete Gamma distribution was used to model evolutionary rate differences among sites [5 categories (+ *G*, parameter = 6.7916)] with some sites allowed to be evolutionarily invariable ([+ *I*], 1.98% sites). There were a total of 1035 positions in the final dataset. Evolutionary analyses were conducted in MEGA X^43,44^.

### Salivary gland gene silencing using double stranded RNA

*Aedes aegypti* salivary gland cDNA was used to amplify dsRNA targets with T7-tagged primers (Table S1). The PCR products were in vitro transcribed using T7 Scribe kit (Cellscript, USA). dsRNA was annealed by heating to 95 °C and slow cooling in a thermocycler. Three to five-day-old adult female mosquitoes were cold-anesthetized and intra-thoracically injected with 2 µg of dsRNA using Nanoject II (Drummond Scientific Company, USA). The same quantity of dsRNA against the bacterial gene *LacZ* was injected as a control (dsCtrl). Four days post dsRNA injection, gene depletion was validated in SG by RT-qPCR.

### Gene expression quantification using real-time quantitative polymerase chain reaction

Total RNA was extracted from 10 SG using E.Z.N.A. Total RNA kit I (Omega Bio-Tek, USA), DNAse treated using Turbo DNA-free kit (Thermo Fisher Scientific, USA), and reverse transcribed using iScript cDNA synthesis kit (Bio-Rad, USA). Gene expression was quantified using qPCR with SensiFast Sybr no-Rox kit (Bioline, USA) and gene specific primers (Table S1). *Actin* expression was used for normalization. The reactions were performed using the following conditions: 95 °C for 10 min, 40 cycles of 95 °C for 5 s, 60 °C for 20 s and melting curve analysis. The 2^−∆∆Cq^ method was used to calculate relative fold changes^45^.

### Quantification of viral genomic RNA (gRNA) copies using RT-qPCR

At 8 days post inoculation, individual pairs of SG were collected in 350 µl of TRK lysis buffer and homogenized at RT for 40 s using a mini beadbeater-96 (Biospec Products, USA) before RNA extraction with E.Z.N.A Total RNA kit I. DENV2 gRNA copies were quantified by RT-qPCR using i-Taq universal probes one-step kit, and ZIKV and CHIKV gRNA copies with i-Taq Universal SYBR green one-step kit with specific primers (Table S2). CFX96 Touch Real-Time PCR Detection System (Bio-Rad, Singapore) was used for amplification with the following thermal profile: 50 °C for 10 min, 95 °C for 1 min, 40 cycles at 95 °C for 10 s, 60 °C for 15 s. A melt-curve analysis was added for the SYBR-based quantification.

To quantify gRNA copies, a standard curve for each qPCR target was generated. qPCR targets were amplified from viral cDNA with the qPCR primers and forward primer tagged with T7. RNA fragments were generated with T7-Scribe kit (Cell Script, USA). RNA target copies were estimated based on Nanodrop quantification and used to generate an absolute standard equation. Three standard dilutions per plate were then added to adjust for inter-plate variation.

### Statistical analyses

Gene expressions were normalized by log2 transformation and gRNA copies by log10 transformation. Differences in normalized values were then tested with unpaired T-test (Microsoft Excel).

## Results

### High-resolution proteomics of salivary glands

A total of 218 proteins with at least two unique peptides (95% confidence per peptide) were identified in uninfected *A. aegypti* SG (Table S3). To identify proteins, we used a custom protein database which included annotated *Aedes* proteins from VectorBase and UniProt, as well as from a de novo assembled transcriptome (unpublished) generated from our previous work. This *A. aegypti* SG transcriptome is specific for the same time point and mosquito colony^15^, unique to our study conditions for the *A. aegypti* Singapore strain. We identified five newly annotated proteins which included one putative-C-type lectin, one 18.6 kDa secreted protein, one uncharacterized (no homolog in *Drosophila melanogaster*) protein, one aggrecan core-like protein and one 34 kDa salivary protein. A large majority (175 out of 218) of the proteins that we detected were also found in the only other high-resolution proteomic analysis of uninfected *A. aegypti* SG^32^ (Fig. S1a). Discrepancies between the studies may stem from the different starting protein amounts, mosquito colonies and mosquito age at collection. Signal peptides (SP), which direct proteins to the secretory pathways^46^, may indicate secretion into saliva. We detected 71 SP-proteins (32.57% of all proteins), among which 39 were previously found in *A. aegypti* saliva^27^ (Fig. S1b). Of note, proteins could also be secreted by non-classical pathways^46,47^ as exemplified by SGS1, which does not have an SP but is detected in saliva^27^. Hereafter, proteins with a SP (including the secreted SGS1) were categorized as secretory proteins, whereas non-SP proteins were considered as cellular proteins.

**Figure 1:**
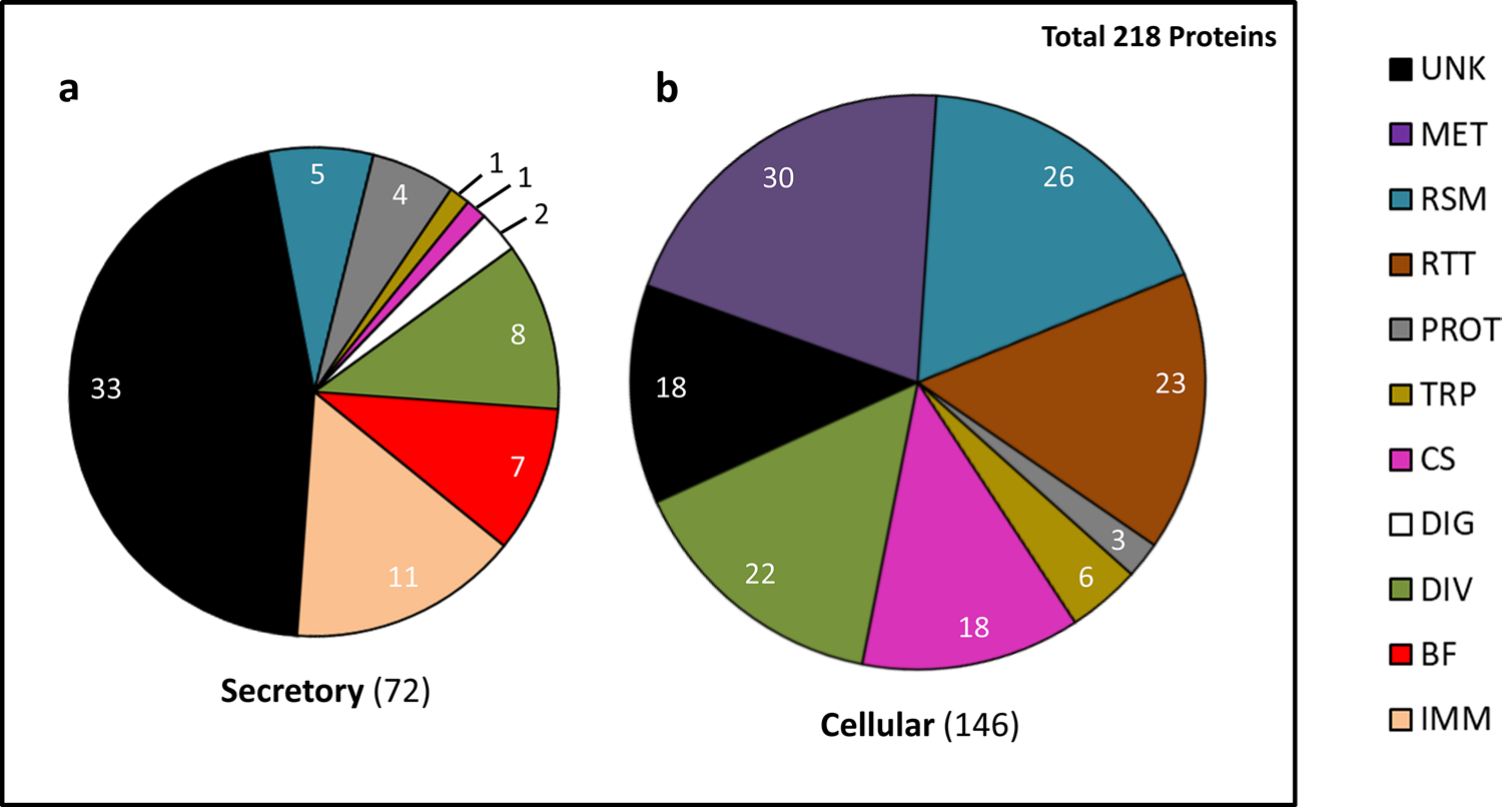
Proteome of uninfected *A. aegypti* SG. (**a**,**b**) Distribution of the putative functions in secretory (identified by a SP, except for SGS1) (**a**) and cellular (**b**) proteins. Numbers of proteins per group are indicated. *UNK* unknown functions, *MET* metabolism, *RSM* redox, stress and mitochondria, *RTT* replication, transcription and translation, *PROT* proteolysis, *TRP* transport, *CS* cytoskeleton and structure, *DIG* digestion, *DIV* diverse functions, *BF* blood feeding, *IMM* immunity.

Among the annotated proteins, 31 had a putative function in ribosome, stress and mitochondria (RSM), 30 in metabolism (MET), 23 in replication, transcription, translation (RTT) and 19 in cytoskeleton (CS). Most of these (97 out of 103) did not have a SP (Fig. 1a,b). There were also seven proteins related to proteolysis (PROT), two related to polysaccharide digestion (DIG), seven related to transport (TRP), 30 related to diverse functions (DIV) and 51 had unknown functions (UNK) (Fig. 1a,b; Table S3). Interestingly, there were 11 proteins related to immunity, including three serpins (SRPNs), one serine protease, four C-type lectins (CTLs), two fibrinogen related protein (FREPs) and one lysozyme (LYS) (Table S3). We did not detect proteins related to signaling of the canonical immune pathways. Seven proteins were related to blood-feeding (BF) and included two D7 proteins, two apyrases, one ADA, a prosialokinin precursor and one odorant binding protein (OBP) (Fig. 1a,b; Table S3). All immunity- and BF-related proteins had a SP (Fig. 1a,b; Table S3).

### Salivary gland proteome response to DENV2, ZIKV or CHIKV infection

**Figure 2:**
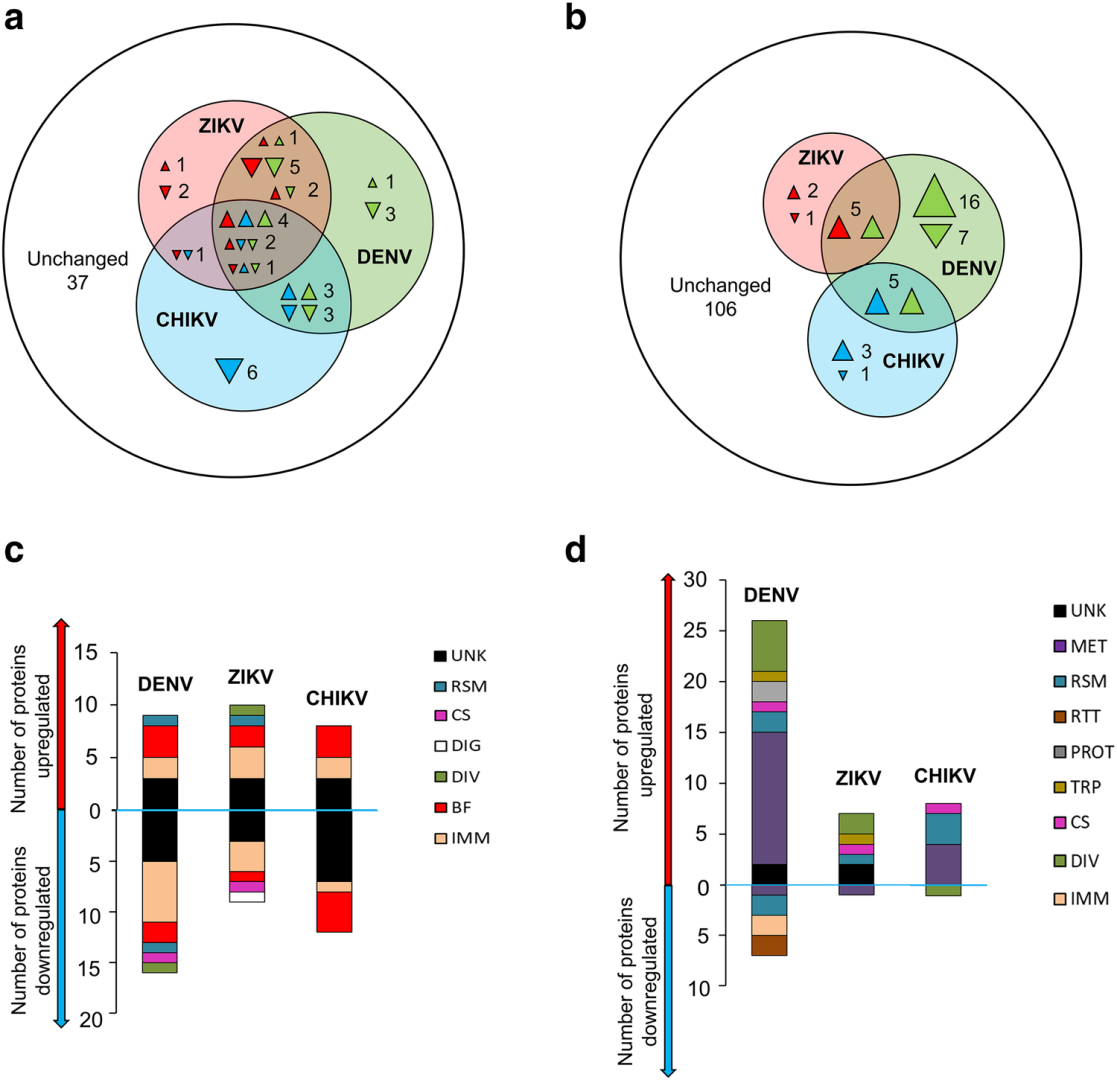
Differentially expressed proteins (DEP) in *A. aegypti* SG upon infection with either DENV2, ZIKV or CHIKV. (**a**,**b**) Overlapping expression of secretory (identified by a SP, except SGS1) (**a**) and cellular (**b**) proteins regulated by DENV2 (green), ZIKV (red) and CHIKV (blue). Colorless area shows number of unregulated proteins. The apex of triangles indicate the direction of regulation for the corresponding color code and triangle size indicates the number of regulated proteins. (**c**,**d**) Functional annotation of secretory (**c**) and cellular (**d**) DEP regulated by DENV2, ZIKV and CHIKV infections. *UNK* unknown functions, *MET* metabolism, *RSM* redox, stress and mitochondria, *RTT* replication, transcription and translation, *PROT* proteolysis, *TRP* transport, *CS* cytoskeleton and structure, *DIG* digestion, *DIV* diverse functions, *BF* blood feeding, *IMM* immunity.

Owing to the different EIP for flaviviruses and alphaviruses^9,11^, SG were dissected at 14 days post oral infection (dpi) for DENV2 and ZIKV, and at seven dpi for CHIKV. We used blood inocula for mosquito oral infections that we previously showed result in 100% of infected SG at the collection time^15^. Controls for DENV2 and ZIKV, and for CHIKV were dissected at the corresponding times post feeding on uninfectious blood. Comparisons were made with the age-matched controls. Using iTRAQ-based quantitative proteomics, we found 35, 17 or 16 upregulated, and 23, 10 or 13 downregulated proteins following DENV2, ZIKV or CHIKV infection, respectively (Fig. 2a,b; Fig. S2, Table S4). We also detected multiple viral proteins, including viral polyproteins (Table S5), in the SG proteome, thereby confirming infection with the corresponding virus.

Seven proteins were commonly regulated by all infections (Fig. 2a,b; Table S4). Among them, the immunity-related protein GILT-like (AAEL004873), the BF-related protein ADA (AAEL026165), two proteins without conserved functional domains named SGS1 (AAEL09993) and SGBAP (AAegL5.3 AAEL019996/ NCBI GenBank Accession No. EAT45119.1^48^, ABF18177.1^49^) were upregulated by all three virus infections (Fig. 2a,b; Table 1). The BF-related protein D7 (AAEL006424) and the immunity-related protein CTL25 (AAEL000556) were upregulated by ZIKV infection (+ 1.7 times and + 1.5 times, respectively) and downregulated by DENV2 (-3.3 times and -2.5 times, respectively) and CHIKV (− 2.5 times for both proteins) infections. Finally, the immunity-related protein SRPN23 (AAEL002704) was increased 2.5 times by CHIKV infection and decreased 2.5 times by DENV2 and 2 times by ZIKV infections (Table S4). All these commonly-regulated proteins except SGS1 have a SP.

**Table 1:** Effects of the commonly-regulated proteins on DENV2, ZIKV and CHIKV infections in salivary glands.

Gene name	Gene ID	Virus	Fold change at the protein level	Effect on the viral infection
Salivary gland broad antiviral protein (SGBAP)	AAEL019996	DENV2	3.9253	Antiviral
		ZIKV	1.6492	Antiviral
		CHIKV	2.7667	Antiviral
Salivary gland surface protein 1 (SGS1)	AAEL009993	DENV2	2.0232	None
		ZIKV	1.7684	Proviral
		CHIKV	3.1224	None
Adenosine deaminase (ADA)	AAEL026165	DENV2	5.0684	None
		ZIKV	1.4967	None
		CHIKV	3.3300	Antiviral
Gamma interferon responsive lysosomal thiol-like (GILT-like)	AAEL004873	DENV2	2.2093	None
		ZIKV	4.6737	Antiviral
		CHIKV	1.7219	None

Among the secretory proteins, immunity and BF related proteins were the most regulated (Fig. 2c; Table S4). Among the immunity proteins, SRPN25 (AAEL007420) increased 2.5 times upon DENV2 infection and 2 times upon CHIKV infection, while SRPN26 (AAEL003182) was increased 2.5 times only by DENV2 infection. CTL16 (AAEL000533) and CTL25 (AAEL00556) decreased 2 times and 2.5 times, respectively, with DENV2 infection and increased 2.1 times and 1.5 times, respectively, with ZIKV infection. CTL21 (AAEL011408) increased 1.7 times upon ZIKV infection only. A C-type lysozyme (LYSP, AAEL009670) and FREP20 (AAEL000726) were reduced 3.3 times and 5 times, respectively, by DENV2 infection and 2 times and 2.5 times, respectively, by ZIKV infection. FREP22 (AAEL000749) was reduced 5 times upon DENV2 infection only. Regulated BF-related proteins included two apyrases (AAEL006347, AAEL006333) upregulated 4.1 times and 2.3 times, respectively, by DENV2 infection and 3.8 times and 2.5 times, respectively, by CHIKV infection; one D7 protein (AAEL007394) downregulated 1.6 times by DENV2 infection and 10 times by CHIKV infection; another D7 protein (AAEL006417) downregulated 2.5 times by CHIKV infection; a prosialokinin precursor (AAEL000229) downregulated 1.6 times by ZIKV infection and 5 times by CHIKV infection; and a 34 kDa salivary protein (AAEL003600) downregulated 2.5 times by DENV2 infection and 3.3 times by CHIKV infection.

Among the cellular proteins, MET-related proteins were the most regulated (Fig. 2d; Table S4). Within the glycolytic pathway, seven proteins were upregulated by DENV2 infection (5.3 times for glucose-6-phosphate isomerase, AAEL012994; 5.2 times for glyceraldehyde-3-phosphate dehydrogenase, AAEL016984; 4.3 times for an enolase, AAEL024228; 3.9 times for a triosephosphate isomerase, AAEL002542; 3.2 times for a phosphoglycerate kinase, AAEL004988; 3.1 times for a fructose-bisphosphate aldolase, AAEL005766; and 2.7 times for a pyruvate kinase, AAEL014913) and two were upregulated by CHIKV infection (7.8 times for glyceroaldehyde-3-phosphate dehydrogenase, AAEL016984; and 5.6 times for a pyruvate kinase, AAEL014913). Within the tricarboxylic acid (TCA) cycle, two proteins were upregulated by DENV2 infection (2.5 times for an aconitase, AAEL012897; 2.4 times for a malate dehydrogenase, AAEL007707), while another malate dehydrogenase (AAEL008166) was upregulated 2.6 times by CHIKV infection. Other proteins related to metabolism were regulated by DENV2 infection and included two proteins related to fatty acid metabolism (+ 2.6 times for a pyruvate carboxylase, AAEL009691; − 2 times for a saposin, AAEL003046), one protein related to energy metabolism (+ 3.9 times for an arginine kinase, AAEL009185) and one related to amino acid metabolism (+ 2.4 times for an aspartate amino transferase, AAEL002399). Another protein related to the amino acid metabolism (pyrroline-5-carboxylate dehydrogenase, AAEL005422) was downregulated 1.6 times by ZIKV infection (Table S4).

Among RSM-related proteins, a protein disulfide isomerase (PDI, AAEL002501) was commonly upregulated by both flaviviral infections, 2.1 times by DENV2 infection and 1.7 times by ZIKV infection. However, another PDI (AAEL000641) was downregulated 1.6 times by DENV2 infection only. DENV2 and CHIKV infections commonly upregulated a thioredoxin reductase (AAEL002886) 1.6 times and 2.1 times, respectively, and 3-ketoacyl-CoA thiolase (AAEL010697) 1.6 times and 2.5 times, respectively. Few other RSM-related proteins were separately regulated by either of the three infections (Table S4). The weak overlap of regulated proteins between the three infections indicates a virus-specific regulation of proteins in SG.

### Effect of the virus-induced proteins on the viral load in salivary glands

**Figure 3:**
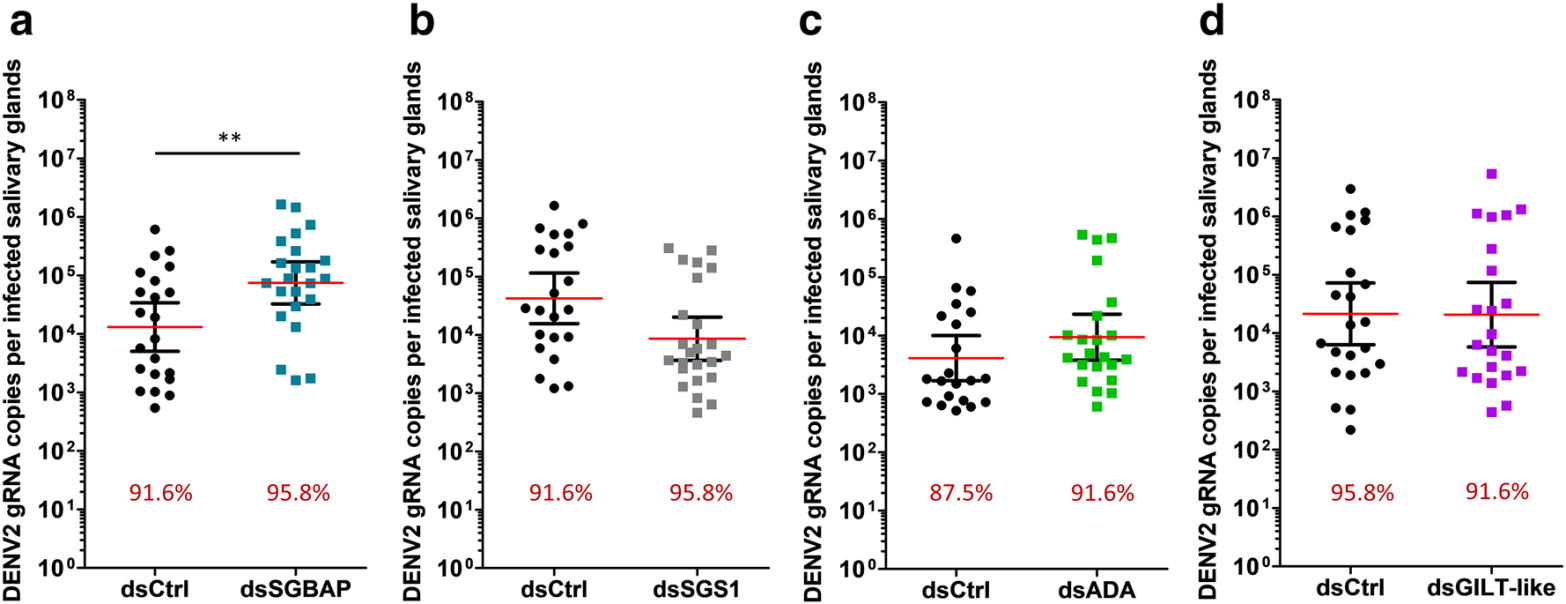
Effect of gene silencing on DENV2 infection in SG. (**a–d**) DENV2 infection level in SG upon silencing of *SGBAP* (**a**), *SGS1* (**b**), *ADA* (**c**), and *GILT*-like (**d**). Infection intensity (defined as gRNA copies per infected SG) is shown as points with lines showing geometric mean ± 95% C.I. from 24 individual pairs of SG. Each dot represents one sample. Infection prevalence is indicated by red numbers. ds*Ctrl*, dsRNA against LacZ; ds*SGBAP*, dsRNA against salivary gland broad spectrum antiviral protein; ds*SGS1*, dsRNA against salivary gland surface protein 1; ds*ADA*, dsRNA against adenosine deaminase protein; ds*GILT-like*, dsRNA against gamma interferon responsive lysosomal thiol protein-like. *p < 0.05; **p < 0.01 as determined by unpaired t-test. Each graph combines results from the same mosquito batch.

**Figure 4:**
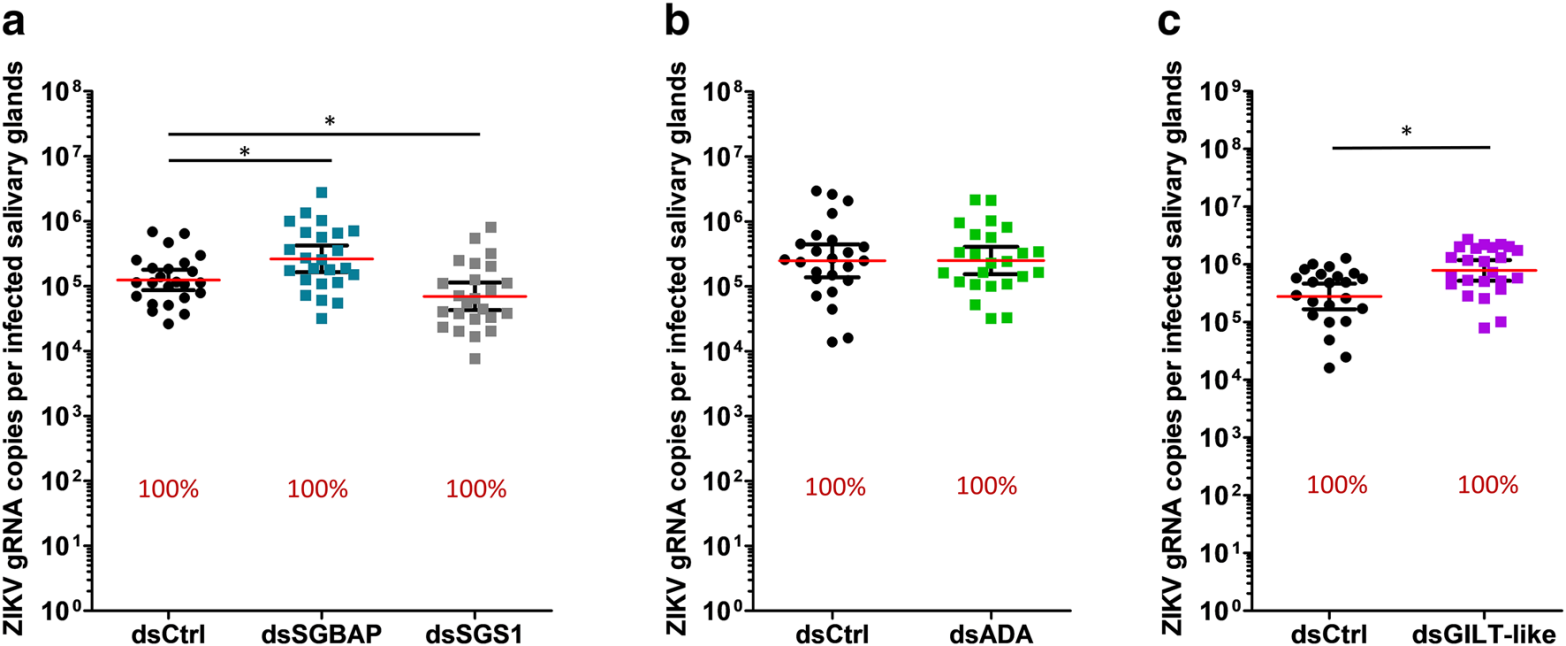
Effect of gene silencing on ZIKV infection in SG. (**a–c**) ZIKV infection level in SG upon silencing of *SGBAP*, *SGS1* (**a**), *ADA* (**b**), and *GILT*-like (**c**). Infection intensity (defined as gRNA copies per infected SG) is shown as points with lines showing geometric mean ± 95% CI from 24 individual pairs of SG. Each dot represents one sample. Infection prevalence is indicated byred numbers. ds*Ctrl*, dsRNA against LacZ; ds*SGBAP*, dsRNA against salivary gland broad spectrum antiviral protein; ds*SGS1*, dsRNA against salivary gland surface protein 1; ds*ADA*, dsRNA against adenosine deaminase protein; ds*GILT-like*, dsRNA against gamma interferon responsive lysosomal thiol protein-like. *p < 0.05; **p < 0.01; determined by unpaired t-test. Each graph combines results from the same mosquito batch.

**Figure 5:**
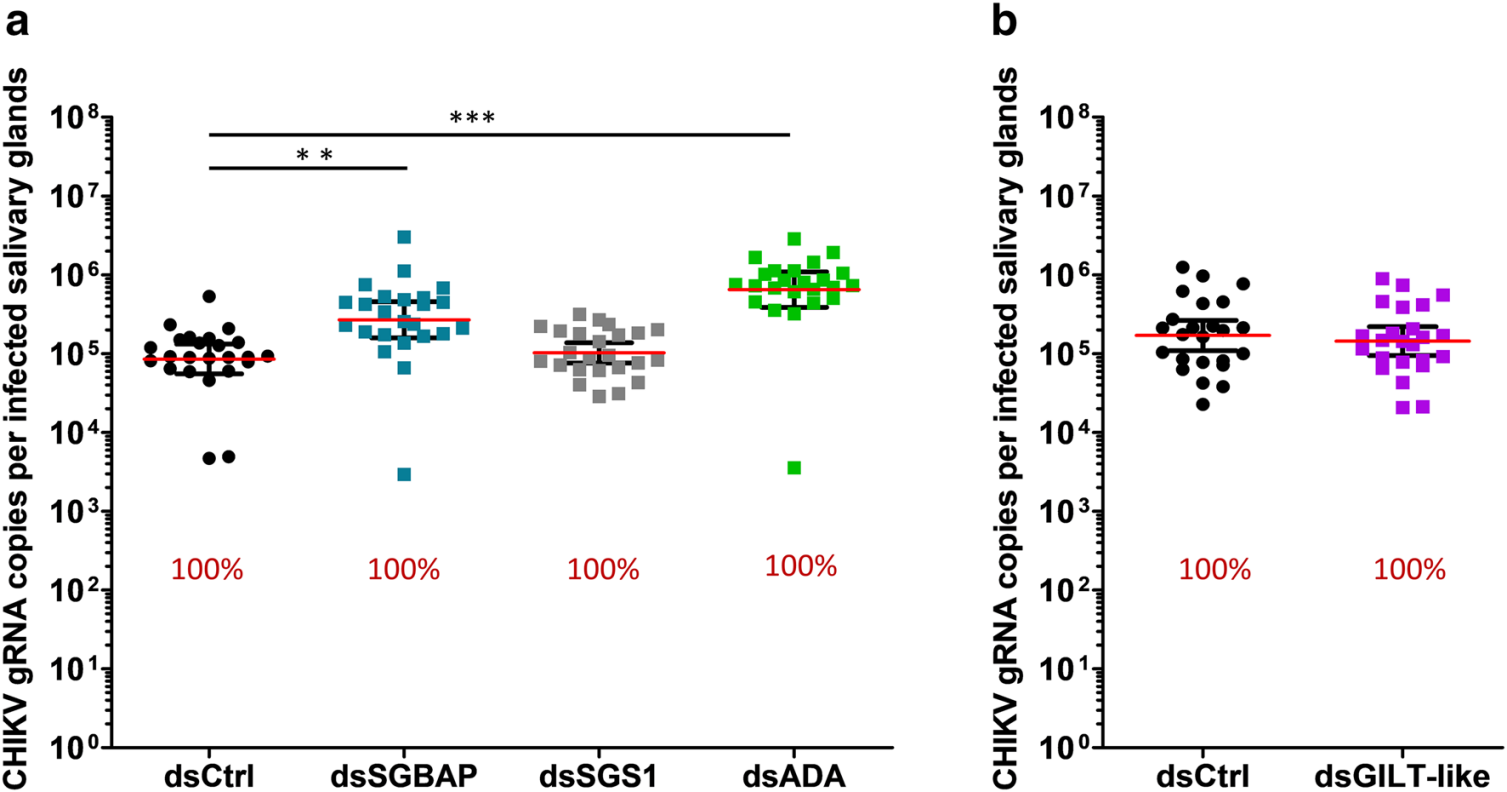
Effect of gene silencing on CHIKV infection in SG. (**a–c**) CHIKV infection level in SG upon silencing of *SGBAP*, *SGS1*, *ADA* (**a**), and *GILT*-like (**b**). Infection intensity (defined as gRNA copies per infected SG) is shown as pointswith lines showing geometric mean ± 95% C.I. from 24 individual pairs of SG. Each dot represents one sample. Infection prevalence is indicated by red numbers. ds*Ctrl*, dsRNA against LacZ; ds*SGBAP*, dsRNA against salivary gland broad spectrum antiviral protein; ds*SGS1*, dsRNA against salivary gland surface protein 1; ds*ADA*, dsRNA against adenosine deaminase protein; ds*GILT-like*, dsRNA against gamma interferon responsive lysosomal thiol protein-like. *p < 0.05; **p < 0.01; determined by unpaired t-test. Each graph combines results from the same mosquito batch.

To evaluate the impact of the four upregulated proteins (i.e., SGBAP, SGS1, ADA and GILT-like) on DENV2, ZIKV and CHIKV infections, we depleted these proteins in SG by RNAi-mediated gene silencing. We studied the upregulated proteins for two reasons: (i) we hypothesized that protein induction indicated an antiviral function although this is not always the case, and (ii) we considered that the effect of the depletion would be stronger for upregulated proteins than for proteins downregulated by the viral infections. DsRNA (dsCtrl) targeting the bacterial gene *LacZ* was injected as control. Depending on the targeted gene, we obtained a silencing efficiency ranging from 48.7 to 73.9% at the mRNA level in SG (Fig. S3). While variations in mRNA quantity may not reflect the variations in protein level, the gene silencing affected the virus load as with previous studies^15^. Furthermore, we considered that the observation of a phenotype (i.e. effect on viral load) indicated that the protein was at least partially depleted. To specifically study the impact of gene depletion in SG, we bypassed the midgut barrier by infecting mosquitoes through intra-thoracic inoculation. We also used a non-saturating inoculum that enabled us to observe an increase or a decrease in the infection level^15^. At 8 days post inoculation (dpin) with DENV2 and ZIKV, and four dpin with CHIKV, we quantified the gRNA in SG. We then calculated the infection prevalence (defined as the percentage of infected SG) and the infection intensity (measured as the viral gRNA copies per infected SG). We used different mosquito batches to test the different genes, and because we observed that infection in control mosquitoes varied between batches (Figs. 3, 4, 5), the infection outputs were compared within the batches.

For all three viruses, infection prevalence was not altered by any gene silencing (Figs. 3, 4, 5). Of note, infection prevalence was 100% for ZIKV and CHIKV, thereby preventing observation of a pro-viral effect with this parameter. Interestingly, gene silencing altered infection intensity in a virus-specific manner. DENV2 infection intensity was increased 3.6 times by SGBAP depletion (Fig. 3). ZIKV infection intensity increased 2.6 times and 2.5 times upon SGBAP and GILT-like depletions, respectively, and decreased 1.31 times upon SGS1 depletion (Fig. 4). CHIKV infection intensity was 3.8 times and 7.5 times higher when SGBAP and ADA were depleted, respectively (Fig. 5). By studying SG proteins with uncharacterized impact on viral infection, we identified the virus-specific function of GILT-like, SGS1 and ADA, and the broad effect of SGBAP in reducing the infections by DENV2, ZIKV and CHIKV (Table 1).

## Discussion

SG proteomic response to infection regulates viral transmission (i) by modulating the mosquito antiviral response, which reduces the virus amount in SG and saliva, and (ii) by altering the production of salivary components, which influences the skin infection. Despite the relevance of SG in transmission, there is a dearth of knowledge about SG response to infection at the proteome level. Leveraging cutting-edge proteomics technology, this study bridges this knowledge gap by describing the SG response to DENV2, ZIKV and CHIKV infections in *A. aegypti* at the global proteome level. Using high-resolution proteomics, we identified 218 proteins expressed in SG with putative functions in immunity, blood-feeding and cellular machinery. Using isobaric tag-based quantitative proteomics, we detected 58 proteins that were regulated by DENV2 infection, 27 by ZIKV infection and 29 by CHIKV infection. While a majority of proteins were not commonly regulated by all three viruses, four proteins were significantly upregulated in SG by DENV2, ZIKV and CHIKV infections. Hypothesizing that their upregulation was related to an antiviral response, we separately tested their functions in SG. The results suggest an antiviral function of GILT-like against ZIKV, ADA against CHIKV and the proviral function of SGS1 for ZIKV. Most interestingly, we showed that SGBAP has a broad antiviral effect on DENV2, ZIKV and CHIKV infections in mosquito SG.

We found that a large majority of the SG proteins related to immunity were regulated by the infections. Among the four CTLs detected in SG, CTL16, CTL21, CTL25 were upregulated by ZIKV infection, while CTL16 and CTL25 were downregulated by DENV2 infection and CTL25 downregulated by CHIKV infection (Fig. S2; Table S4). CTL16 and CTL25 were previously detected in SG^50,51^. CTLs are soluble proteins with carbohydrate binding activity and have multiple functions in regulating pathogen infection^52^. Studies have shown that CTLs can facilitate arbovirus attachment and entry into cells, as well as enhance infection. For example, galactose-binding CTL1 (mos-*GCTL1*, AAEL000563) recruitment by protein tyrosine phosphatase-1 facilitates West Nile Virus attachment and cell entry^53^, CTL3 (mos-*GCTL3*, AAEL029058) interacts with DENV2 envelop to enhance viral infection^54^ and mosquito CTL4 (AGAP005335) and CTLMA2 (AGAP005335) are required for clearance of *Escherichia coli*. These studies suggest a role for CTL in immunity^55^. Both FREP20 and FREP22 expressed in SG were downregulated by DENV2 infection, while only FREP20 was downregulated by ZIKV (Fig. S2; Table S4). FREPs, also called immunolectins, are pattern recognition receptors, which activate innate immune pathways^56^. Among the three SRPNs identified in SG, SRPN 23 was downregulated by both DENV2 and ZIKV infections, but upregulated by CHIKV infection (Fig. S2; Table S4). SRPN 25 was upregulated by both DENV2 and CHIKV infections, and SRPN 26 was upregulated by DENV2 infection alone (Fig. S2; Table S4). SRPNs regulate innate immunity by inhibiting protease signaling cascade^57^. For this, they bind trypsin-like targets through an arginine or lysine residue at P1 position^58^. Of note, the three SG SRPNs lack the characteristic inhibitory sequence and could therefore be non-inhibitory or act in a non-classical way as protease inhibitors^49,58^. The one LYS (LYSC9) expressed in SG was downregulated by both DENV2 and ZIKV infections (Fig. S2; Table S4). Its closest *D. melanogaster* homolog (i.e., LYSP) is specifically expressed in SG^59^, while another LYSC in *A. aegypti* was upregulated in midgut by DENV2 infection^60^. LYS have functions in both digestion and immunity^61^. Overall, we identified immunity-related proteins regulated by DENV2, ZIKV or CHIKV infection in SG. Determining their roles in SG immune response will require functional characterization.

We also observed that SG infection influenced the expression of proteins expectorated in saliva. ADA was upregulated by each of the three viruses (Table 1, Fig. S2). Saliva ADA can convert adenosine into inosine at the bite site, thereby inhibiting inflammatory cytokines to prevent the peripheral pain signalling^62^. ADA also enhances DENV2 infection in vitro by inhibiting type I IFN response in human keratinocytes^63^. Both apyrases detected in SG were upregulated by DENV2 and CHIKV (Fig. S2; Table S4). Apyrases are SG-specific proteins that when secreted in saliva can prevent clot formation at the bite site by inhibiting ATP- and ADP-mediated platelet aggregation^64,65^. Accordingly, apyrase content in mosquito SG is inversely proportional to probing time^66^. D7 proteins are highly abundant in mosquito saliva^50,67,68^ and function as scavengers of biogenic amines^69^, which induce vasoconstriction, platelet-aggregation and pain signaling^69^. Interestingly, immunization with recombinant D7 protein from *Culex tarsalis* enhances mortality with West Nile virus in mice^70^. Among the five D7 proteins we identified in *A. aegypti* SG, three were regulated by at least one of the virus infections (Fig. S2; Table S4). A 37 kDa D7 long protein (AAEL006424) was downregulated by both DENV2 and CHIKV infections and upregulated by ZIKV infection (Fig. S2; Table S4). This D7 long form interacts with DENV2 envelope to inhibit infection in vertebrates^29^. Another D7 long protein (AEL006417) was downregulated by both DENV2 and CHIKV infection, while a D7 short protein (AAEL007394) was only downregulated by CHIKV infection alone (Fig. S2; Table S4). Prosialokinin that was downregulated by ZIKV infection (Fig. S2; Table S4) is the precursor of secreted Sialokinin I and II, which have vasodilatory properties^22^. While OBP22 was identified in SG (Table S3), its expression was not altered by any infection. OBPs are soluble ligand binding proteins with high affinity towards hydrophobic odorants and pheromones. They are involved in perception of odor and chemosensory signals, which regulate host-seeking behavior^71^. OBP22 was previously found to be a ligand for fatty acids^72^ and required for efficient biting^16^. Our data suggests that SG infection can modulate transmission by altering saliva composition. Moreover, we reported regulation of proteins related to digestion, metabolism and redox that are discussed in supplemental (S1 Text).

A large proportion of SG proteins that were regulated by the infections remain uncharacterized. In this study, we determined the impact of four uncharacterized infection-responsive proteins in SG infection, i.e., ADA, SGS1, GILT-like and SGBAP (Table 1). Tissue expression analysis based on available *A. aegypti* transcriptome literature^50,73–76^ showed that SGBAP and SGS1 are specifically expressed in SG (Fig. S4). ADA is highly expressed in SG but is also present at lower levels in female abdominal tips (defined as the three terminal abdominal fragments, including genitalia and ovipositor)^74^. GILT-like protein is expressed in a wide range of tissues. While all the four proteins were upregulated by DENV2, ZIKV and CHIKV in SG in the current study, we observed that ADA and GILT-like proteins had virus-specific antiviral properties against CHIKV and ZIKV, respectively, that SGS1 protein had virus-specific proviral function for ZIKV, and that SGBAP protein had broad-spectrum antiviral properties as it reduced infection by all the three viruses. ADA is indirectly involved in immune regulation, as it degrades adenosine, which suppresses immune response^77^. Accordingly, ADA enhances DENV2 infection in keratinocyte cells by inhibiting type I interferon response^77^. ADA levels in SG may thus regulate the balance between immune activation and repression. GILT-like was originally discovered as an interferon-inducible gene in mammals and subsequent characterization revealed its role in antigen presentation, bacterial infection and production of reactive oxygen species^78^, which provides a rationale for its antiviral function. In mosquitoes, GILT-like interacts with *Plasmodium* parasites and limits the parasite motility in skin when expectorated during biting^79^. SGS1 is secreted in saliva through a non-classical pathway^80^ and is a potential receptor for cell entry of avian malaria sporozoites in *A. aegypti* SG^81^. Its proviral effect for ZIKV might be related to a similar mechanism.

Factors with broad antiviral properties are of particular interest in the design of transmission blocking interventions. We revealed the antiviral effect of SGBAP against two flaviviruses and one alphavirus in SG of *A. aegypti.* SGBAP does not contain conserved functional domains as determined from searches of NCBI conserved domain, InterPro—EMBL-EBI and PROSITE-Expasy databases, and has no homolog in *D. melanogaster*, making it difficult to speculate on its structure and mechanism of action. SGBAP is a small protein of 130 amino acid residues (mature form) (we suggest a re-annotation of the gene in S1 text and Fig. S5) and is secreted in *A. aegypti* saliva^27^. An earlier transcriptomic study suggested it originated from a truncation of a gene from the 34 kDa protein family^49^. To identify SGBAP-related genes in other arbovirus mosquito vectors, we built a phylogenetic tree based on cDNA sequences (Fig. S6). While we searched all mosquito species available on VectorBase, *SGBAP* had only 21 putative homologs in *A. aegypti*, *Aedes albopictus* and *Culex quinquefasciatus* with relatively low bootstraps. However, based on amino acid identity, SGBAP closest match is an *A. albopictus* protein (AALF004420, 56% identity) with unspecified function. Functional homology among SGBAP orthologues should be experimentally tested. In *A. aegypti*, SGBAP broad antiviral effect warrants further studies to understand its mechanism in SG and its function in saliva, where it could inhibit virus propagation.

In conclusion, we expanded the understanding of the SG response to DENV2, ZIKV and CHIKV infections by using high-resolution quantitative proteomics in mosquito SG. We also identified new antiviral factors in SG, shedding new light on the antiviral response, which can be used to promote transmission blocking interventions.

## Supplementary Information

Supplementary Information 1.

Supplementary Figure S1.

Supplementary Figure S2.

Supplementary Figure S3.

Supplementary Figure S4.

Supplementary Figure S5.

Supplementary Figure S6.

Supplementary Table S3.

Supplementary Table S4.

Supplementary Table S5.
