# Supplementary material for: High resolution proteomics of Aedes aegypti salivary glands infected with either dengue, Zika or chikungunya viruses identify new virus specific and broad antiviral factors
Source: Sci Rep. 2021 Dec 8;11:23696. doi: 10.1038/s41598-021-03211-0 (PMC8654903; doi:10.1038/s41598-021-03211-0)
Supplement: Supplementary file 1 — Supplementary Information 1. [file 41598_2021_3211_MOESM1_ESM.docx]

**Supplementary information**

**S1 Text**

**DEPs related to digestion**

DENV2 infection of human foreskin fibroblasts requires glycolysis^1^. Upregulation of several glycolytic pathway- and tricarboxylic acid cycle (TCA)-related proteins in *A. aegypti* SG (Table S4), primarily by DENV2 infection, suggest similar requirements in mosquitoes. Alternatively, glycolytic enolase (upregulated in this study) is a DENV2 receptor in mosquito cells^2^.

**DEPs related to metabolism**

DENV2-induced expression of pyruvate carboxylase, involved in *de novo* fatty acid (FA) synthesis^3^, and reduced expression of saposin, involved in FA catabolism^4^, suggest that virus multiplication alters host lipid metabolism^5,6^. Aspartate amino transferase, the key enzyme in amino acid metabolism, was upregulated by DENV2 infection in SG as previously observed in dengue patients^7^.

**DEPs related to ribosome stress and mitochondria (RSM)**

The modulation of several RSM-related proteins by all the three viruses suggests a disruption of intracellular redox homeostasis in response to infection. PDIs are essential components of endoplasmic reticulum (ER) and emerge as redox chaperone proteins that play key roles in pathogen attachment-internalization, antigen processing in the ER/phagosome, and regulation of reactive oxygen species production by NADPH oxidases^8,9^. Inhibition of PDI suppresses viral replication and production during antibody dependent enhancement of DENV2 infection in human monocytic cells^9^. Finally, increased expression of enzymatic anti-oxidant, TPX2^10^, by CHIKV infection implies a defensive action to protect oxidative damage.

**Re-annotation of SGBAP (AAEL019996) on VectorBase AAegL 5.3**

Re-annotation of AAEL019996 nucleotide sequence were made to accommodate all theoretical and experimental observations.

Translation initiation site (TIS): TIS can be predicted by correct Kozak sequence G/AXXATGG. The current predicted TIS for has cXXATGG sequence (highlighted in teal blue, Fig. S5), while TIS starting at position 59 (highlighted in purple, Fig. S5) shows ideal Kozak sequence AXXATGG^11,12^. Further, signal peptide (SP) cannot be predicted from current protein sequence starting MDSAA (highlighted in grey, Fig. S5), whereas SP can be predicted from the re-annotated protein sequence (highlighted in red, Fig. S5) starting from re-annotated MVALG (highlighted in grey, Fig. S5) using SignalP 5.0. This TIS was also predicted in an earlier study^13^.

Prediction of intron: The predicted intron (highlighted in yellow, Fig. S5) does not have canonical splice sites, i.e. it has neither the GT at the 5’ nor AG at the 3’ end^14,15^. It also lacks the cis-acting sequence, a polypyrimidine track at the 3’ end of the intron needed for splicing^16^. Thus, the intron is incorrectly annotated. In addition, mRNA sequencing supports the presence of the transcripts that fit the re-annotated sequence (NCBI GenBank accession No: DQ440144.1 and DQ440022.1)^13^ and the authors identified one of them (DQ440144.1) as encoding a truncated 34 kDa family protein. Without this intron, SGBAP protein sequence continues as shown and the re-annotated sequence supported by the mass spectral data.

Mass spectrometry data: Our MS/MS data identified two unique peptides in the current sequence and three peptides in the re-annotated protein sequence (highlighted in green, Fig. S5). The third peptide is located in the re-annotated protein sequence, providing direct experimental evidence for the re-annotated sequence. The re-annotated protein, but not the current predicted protein, contains a second Cys residue (position 113, Fig. S5), which may form a disulfide bond with the other Cys (position 67, Fig. S5).

**Supplementary figure legends**

**Fig. S1.** Comparisons of *A. aegypti* SG proteome between our dataset and two previous studies. (**a**) Venn diagram between SG proteins detected in (i) Singapore *A. aegypti* colony (this study) and (ii) India *A. aegypti* colony^17^. In the comparison, we only used the 721 proteins from *Dhawan et al.* 2017^17^ with at least two unique peptides. (**b**). Venn diagram between (i) SG proteins in Singapore *A. aegypti* colony (this study) and (iii) saliva protein in Rockefeller *A. aegypti* colony^18^.

**Fig. S2**. Heat map showing protein regulations in response to DENV2, ZIKV and CHIKV infections in SG. Differentially expressed proteins were considered when fold change relative to control was above 1.5 or below 0.67.

**Fig S3.** Silencing efficiencies in SG. Each mosquito was injected with dsRNA against the candidate gene. Same quantity of dsCtrl was injected in control mosquitoes. Four days later, mRNA was quantified using RT-qPCR in three pools of 10 SG. *Actin* expression was used for normalization. **a-d**. Silencing efficiency for *SGBAP* (**a**), *SGS1* (**b**), *ADA* (**c**) and *GILT*-like (**d**) in SG upon infection with DENV2. **e-h**. Silencing efficiency for *SGBAP* (**e**), *SGS1* (**f**), *ADA* (**g**) and *GILT*-like (**h**) in SG upon infection with ZIKV. **i-l**. Silencing efficiency for *SGBAP* (**i**), *SGS1* (**j**), *ADA* (**k**) and *GILT*-like (**l**) in SG upon infection with CHIKV. ds*Ctrl*, dsRNA against LacZ; ds*SGBAP*, dsRNA against salivary gland broad spectrum antiviral protein; ds*SGS1*, dsRNA against salivary gland surface protein 1; ds*ADA*, dsRNA against adenosine deaminase protein; ds*GILT-like*, dsRNA against gamma interferon responsive lysosomal thiol protein-like. Bars show means ± s.e.m. from three repeats.

**Fig. S4**. Tissue expression analysis for *SGBAP* (AAEL019996), *SGS1* (AAEL009993), *ADA* (AAEL026165) and *GILT*-like (AAEL004873) genes. Transcripts per million (TPM) in different *A. aegypti* tissues were retrieved using VectorBase from Raquin et al.^19^, Mathews et al.^20^, Anglero-Rodriguez et al.^21^, Dong et al.^22^ and Ribeiro et al.^13^.

**Fig. S5.** Re-annotation of SGBAP (AAEL019996) protein sequence. Current nucleotide and amino acid sequences from VectorBase are shown in top two rows, while sequences with our proposed reannotation are shown in bottom two rows. The translation initiation site (TIS) and the respective Kozak sequences in the current and re-annotated protein sequences are highlighted in teal blue and purple. The signal peptides in the current and re-annotated protein sequences are shown in grey and brown boxes, respectively. The predicted intron sequence (highlighted in yellow) does not have correct donor and acceptor sites as well as polypyrimidine track and hence, was re-annotated as part of the exon. The re-annotation identifies the second Cys that may form the disulphide bridge with other Cys residue (red fonts). The unique peptide sequences detected in our iTRAQ MS/MS analysis are highlighted in green. The arrowheads indicate potential *N*-glycosylation sites. The identification of the C-terminal peptide indicates that the *N*-glycosylation site at the C-terminal is either not or partially glycosylated.

**Fig. S6.** Phylogenetic tree of *SGBAP* (AAEL019996) with 21 homologs in *Aedes aegypti*), *Aedes albopictus*and *Culex quinquefasciatus*. A bootstrap consensus tree inferred from 1000 replicates and is taken to represent the evolutionary history of the taxa analysed. Bootstraps are noted at each node. AAEL and CPI denotes *A. aegypti* and *C. quinquefasciatus* VectorBase gene accession numbers, respectively; Both AALF and LOC^23^ denote *A. albopictus* gene IDs from VectorBase .

**Table legends**

**Table S1.** Primer sequences for RNAi silencing and RT-qPCR quantification

| **Gene name/ Acc. No.** | **dsRNA primers** | **RT-qPCR primers** |
| --- | --- | --- |
| *Actin* / AAEL011197 |  | Fw: GAACACCCAGTCCTGCTGACA  Rv: TGCGTCATCTTCTCACGGTTAG |
| *Lac Z* | Fw: TACCCGTAGGTAGTCACGCA  Rv: TACGATGCGCCCATCTACAC |  |
| *ADA*/ AAEL005672 | Fw: GGTATCACATGGGCCACAAT  Rv: ATTCCGGATGATTCGATTTG | Fw: TGCTTGACCGTTTCGGACAG Rv: CAGTGCTGCCCAAAGCCATA |
| *GILT-like*/ AAEL004873 | Fw: AGTTGAGTTTGAACGGTCCC  Rv: GTCCGGACAGAGCGATTC | Fw: TTTCCGTCAGACCTTCGACA  Rv: CCAAAGTCAGCAGACCCAAC |
|  |  |  |
| *SGS1/* AAEL009993 | Fw: CTTCTGGAAGCCGGAGGT  Rv: CACCACTCTTCACCAACACAA | Fw: ACAAAGTCGGACATCCAGGT Rv: TAGACCACCACGTGCTTCTT |
| *SGAP/* AAEL019996 | Fw: ATTCCACAAAGATGGTCGCT  Rv: CTGCCGAGCCATTACTTCTC | Fw: TTCGCTTGGAATAAATCGTAGTCTG  Rv: TATTCATCATCCATGTAGTTCCCAC |

**Table S2.** Primer sequences for virus quantification

| **Virus name** | **RT-qPCR primers** |
| --- | --- |
| CHIKV | Fw: AAAGGGCAAACTCAGCTTCAC  Rv: GCCTGGGCTCATCGTTATTC |
| DENV2 | Fw: CAGGTTATGGCACTGTCACGAT  Rv:  CCATCTGCAGCAACACCATCTC  Probe:/5HEX/CTCTCCGAGAACAGGCCTCGACTTAAA/3BHQ1/ |
| ZIKV | Fw: CCGCTGCCCAACACAAG  Rv: CCACTAACGTTCTTTTGCAGACAT |

**Table S3.** Proteins identified in uninfected *A. aegypti* SG

**Table S4.** Differentially expressed proteins in *A. Aegypti* SG in response to DENV2, ZIKV and CHIKV infections

**Table S5. List of viral proteins identified in infected SG**

**Supplementary references**

1. Fontaine, K. A., Sanchez, E. L., Camarda, R. & Lagunoff, M. Dengue virus induces and requires glycolysis for optimal replication. *J. Virol.* **89**, 2358–2366 (2015).

2. Muñoz, M. de L. *et al.* Proteomic identification of dengue virus binding proteins in *Aedes aegypti* mosquitoes and *Aedes albopictus* Cells. *BioMed Research International* **2013** e875958 (2013).

3. Park, S.-S., Kim, S.-J., Choi, H., Chang, C. & Kim, E. TR4 promotes fatty acid synthesis in 3T3-L1 adipocytes by activation of pyruvate carboxylase expression. *FEBS Lett.* **588**, 3947–3953 (2014).

4. Geoghegan, V. *et al.* Perturbed cholesterol and vesicular trafficking associated with dengue blocking in Wolbachia -infected *Aedes aegypti* cells. *Nat. Commun.* **8**, 526 (2017).

5. Martín-Acebes, M. A., Blázquez, A.-B., Jiménez de Oya, N., Escribano-Romero, E. & Saiz, J.-C. West Nile virus replication requires fatty acid synthesis but is independent on phosphatidylinositol-4-phosphate lipids. *PLoS One* **6**, (2011).

6. Vial, T. *et al.* Dengue virus reduces AGPAT1 expression to alter phospholipids and enhance infection in Aedes aegypti. *PLoS Pathog.* **15**, e1008199 (2019).

7. Lee, L. K. *et al.* Clinical relevance and discriminatory value of elevated liver aminotransferase levels for dengue severity. *PLoS Negl. Trop. Dis.* **6**, e1676 (2012).

8. Stolf, B. S. *et al.* Protein disulfide isomerase and host-pathogen interaction. *Sci. World J.* **11**, 1749–1761 (2011).

9. Rawarak, N., Suttitheptumrong, A., Reamtong, O., Boonnak, K. & Pattanakitsakul, S. Protein disulfide isomerase inhibitor suppresses viral replication and production during antibody-dependent enhancement of dengue virus infection in human monocytic cells. *Viruses* **11**, 155 (2019).

10. Radyuk, S. N., Klichko, V. I., Spinola, B., Sohal, R. S. & Orr, W. C. The peroxiredoxin gene family in *Drosophila melanogaster*. *Free Radic. Biol. Med.* **31**, 1090–1100 (2001).

11. Kozak, M. Point mutations define a sequence flanking the AUG initiator codon that modulates translation by eukaryotic ribosomes. *Cell* **44**, 283–292 (1986).

12. Kozak, M. Possible role of flanking nucleotides in recognition of the AUG initiator codon by eukaryotic ribosomes. *Nucleic Acids Res* **9**, 5233-5252 (1981).

13. Ribeiro, J. M. *et al.* An annotated catalogue of salivary gland transcripts in the adult female mosquito, *Aedes aegypti*. *BMC Genomics* **8**, 6 (2007).

14. Breathnach, R. & Chambon, P. Organization and expression of eucaryotic split genes coding for proteins. *Annu. Rev. Biochem.* **50**, 349–383 (1981).

15. [Breathnach](https://pubmed-ncbi-nlm-nih-gov.libproxy1.nus.edu.sg/?term=Breathnach+R&cauthor_id=283395) R,  [Benoist](https://pubmed-ncbi-nlm-nih-gov.libproxy1.nus.edu.sg/?term=Benoist+C&cauthor_id=283395) C, [O'Hare](https://pubmed-ncbi-nlm-nih-gov.libproxy1.nus.edu.sg/?term=O%27Hare+K&cauthor_id=283395) K,  [Gannon](https://pubmed-ncbi-nlm-nih-gov.libproxy1.nus.edu.sg/?term=Gannon+F&cauthor_id=283395) F, [Chambon](https://pubmed-ncbi-nlm-nih-gov.libproxy1.nus.edu.sg/?term=Chambon+P&cauthor_id=283395) P. Ovalbumin gene: evidence for a leader sequence in mRNA and DNA sequences at the exon-intron boundaries. *Proc Natl Acad Sci* **75**, 4853-7 (1978).

16. Coolidge, C. J., Seely, R. J. & Patton, J. G. Functional analysis of the polypyrimidine tract in pre-mRNA splicing. *Nucleic Acids Res.* **25**, 888–896 (1997).

17. Dhawan, R. *et al.* Mosquito-borne diseases and omics: salivary gland proteome of the Female *Aedes aegypti* mosquito. *Omics J. Integr. Biol.* **21**, 45–54 (2017).

18. Sun, P. *et al.* A mosquito salivary protein promotes flavivirus transmission by activation of autophagy. *Nat. Commun.* **11**, 260 (2020).

19. Raquin, V. *et al.* Individual co-variation between viral RNA load and gene expression reveals novel host factors during early dengue virus infection of the *Aedes aegypti* midgut. *PLoS Negl. Trop. Dis.* **11**, e0006152 (2017).

20. Matthews, B. J., McBride, C. S., DeGennaro, M., Despo, O. & Vosshall, L. B. The neurotranscriptome of the *Aedes aegypti* mosquito. *BMC Genomics* **17**, 32 (2016).

21. Angleró-Rodríguez, Y. I. *et al.* *Aedes aegypti* molecular responses to Zika virus: modulation of infection by the toll and JAK/STAT immune pathways and virus host factors. *Front. Microbiol.* **8**, (2017).

22. Dong, S., Behura, S. K. & Franz, A. W. E. The midgut transcriptome of *Aedes aegypti* fed with saline or protein meals containing chikungunya virus reveals genes potentially involved in viral midgut escape. *BMC Genomics* **18**, (2017).

23. Palatini, U. *et al.* Improved reference genome of the arboviral vector *Aedes albopictus*. *Genome Biol.* **21**, 215 (2020).
