# Supplementary figures and images for: High resolution proteomics of Aedes aegypti salivary glands infected with either dengue, Zika or chikungunya viruses identify new virus specific and broad antiviral factors
Source: Sci Rep. 2021 Dec 8;11:23696. doi: 10.1038/s41598-021-03211-0 (PMC8654903; doi:10.1038/s41598-021-03211-0)

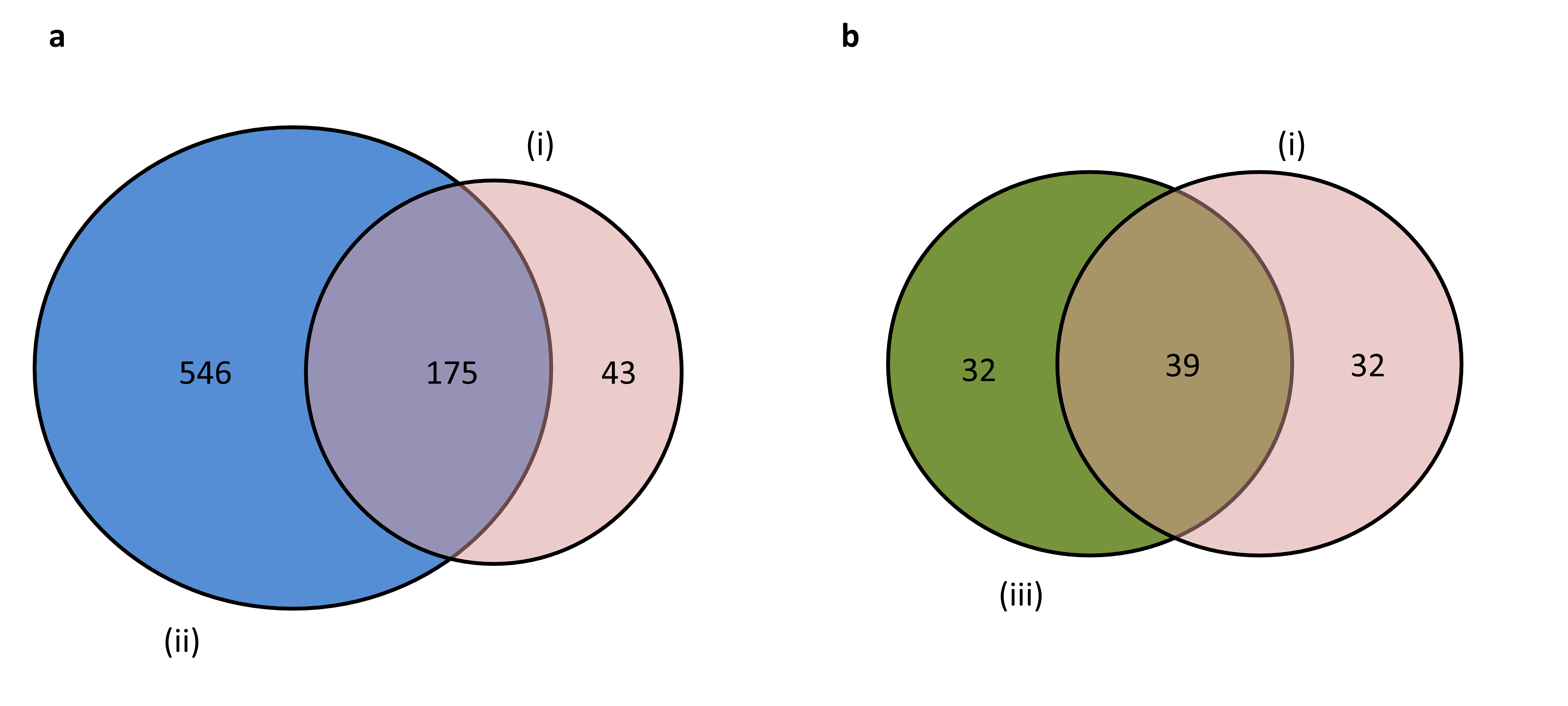

Supplement: Supplementary file 2 — Supplementary Figure S1. [file 41598_2021_3211_MOESM2_ESM.tif]

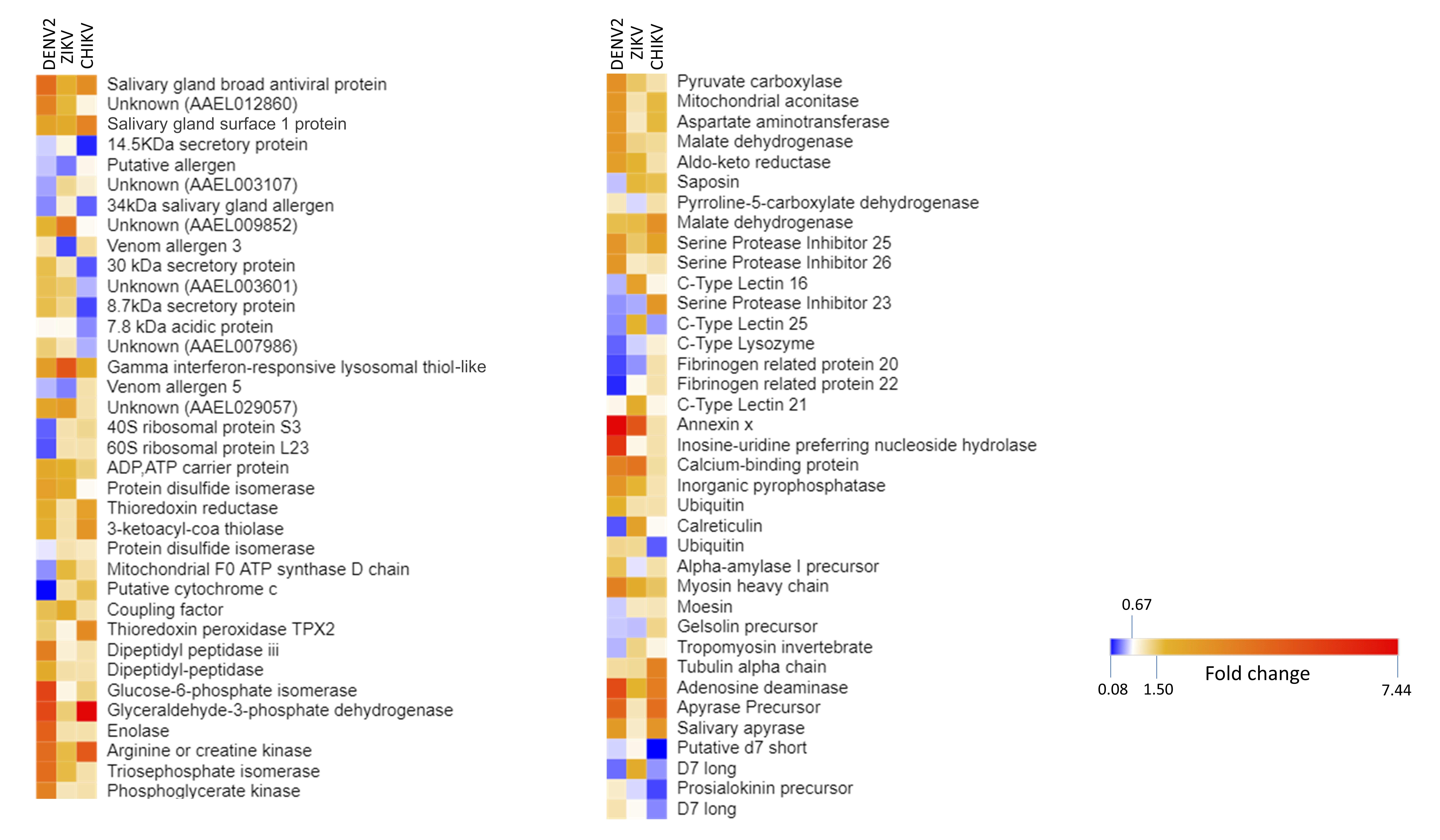

Supplement: Supplementary file 3 — Supplementary Figure S2. [file 41598_2021_3211_MOESM3_ESM.tif]

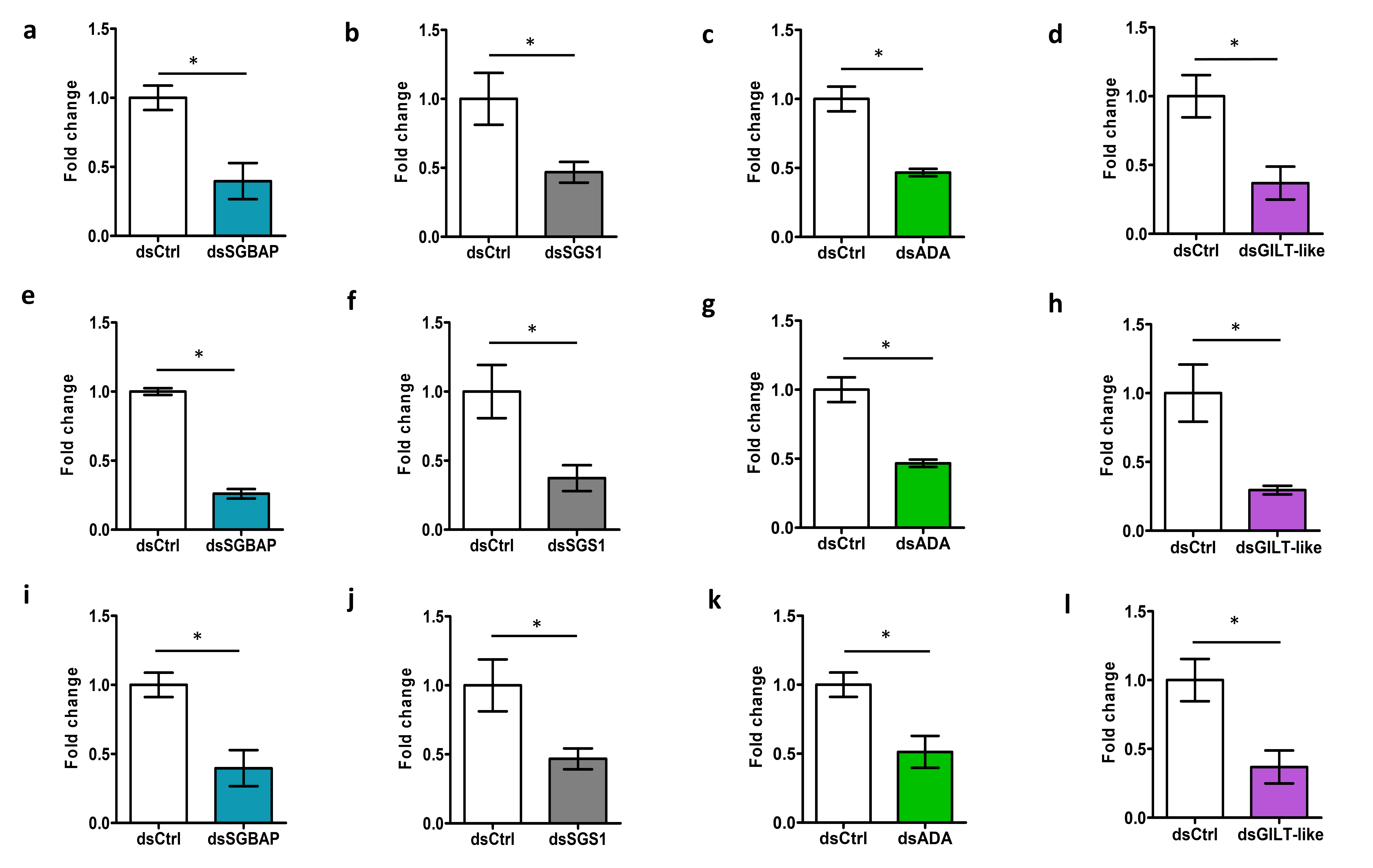

Supplement: Supplementary file 4 — Supplementary Figure S3. [file 41598_2021_3211_MOESM4_ESM.tif]

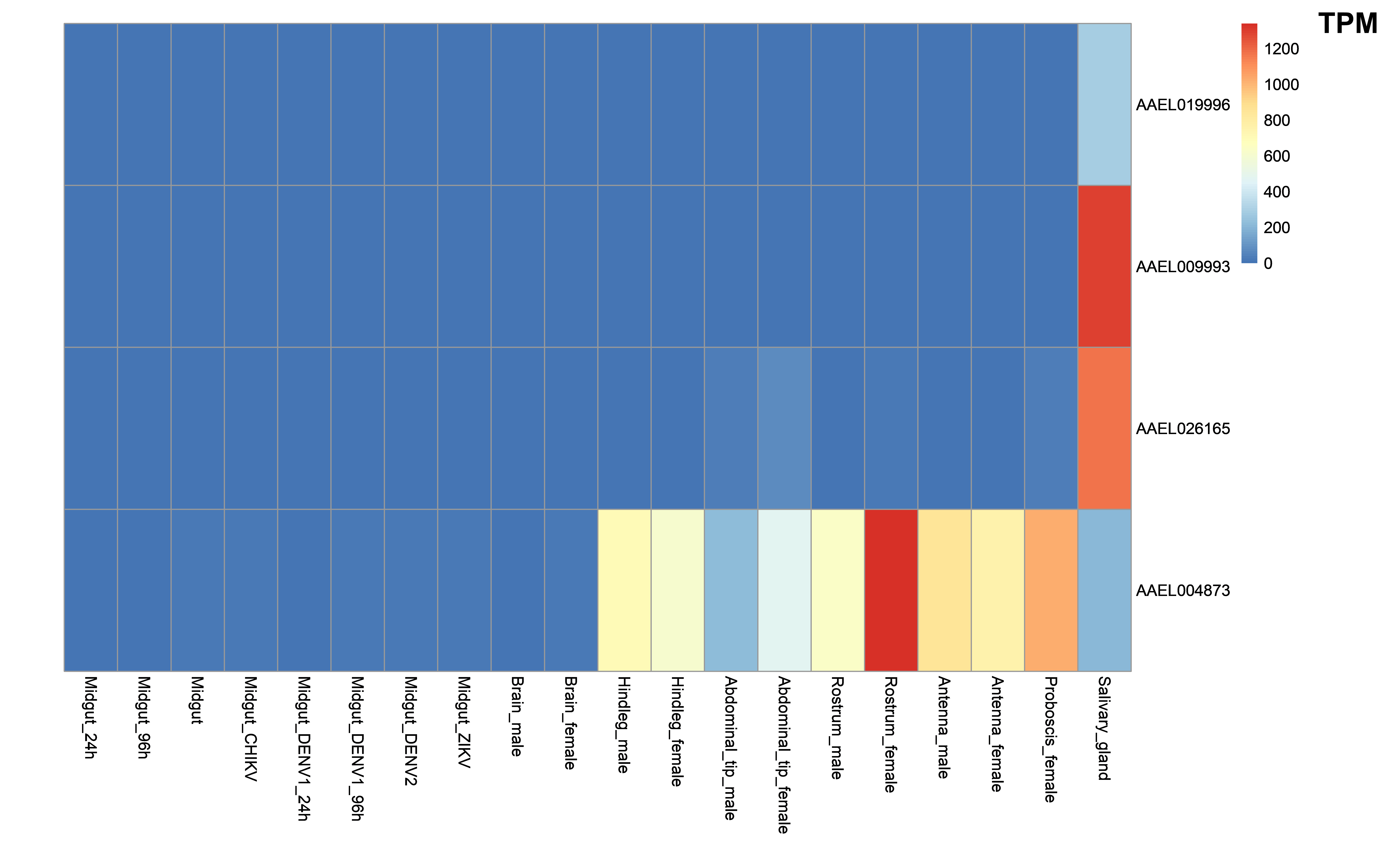

Supplement: Supplementary file 5 — Supplementary Figure S4. [file 41598_2021_3211_MOESM5_ESM.tif]

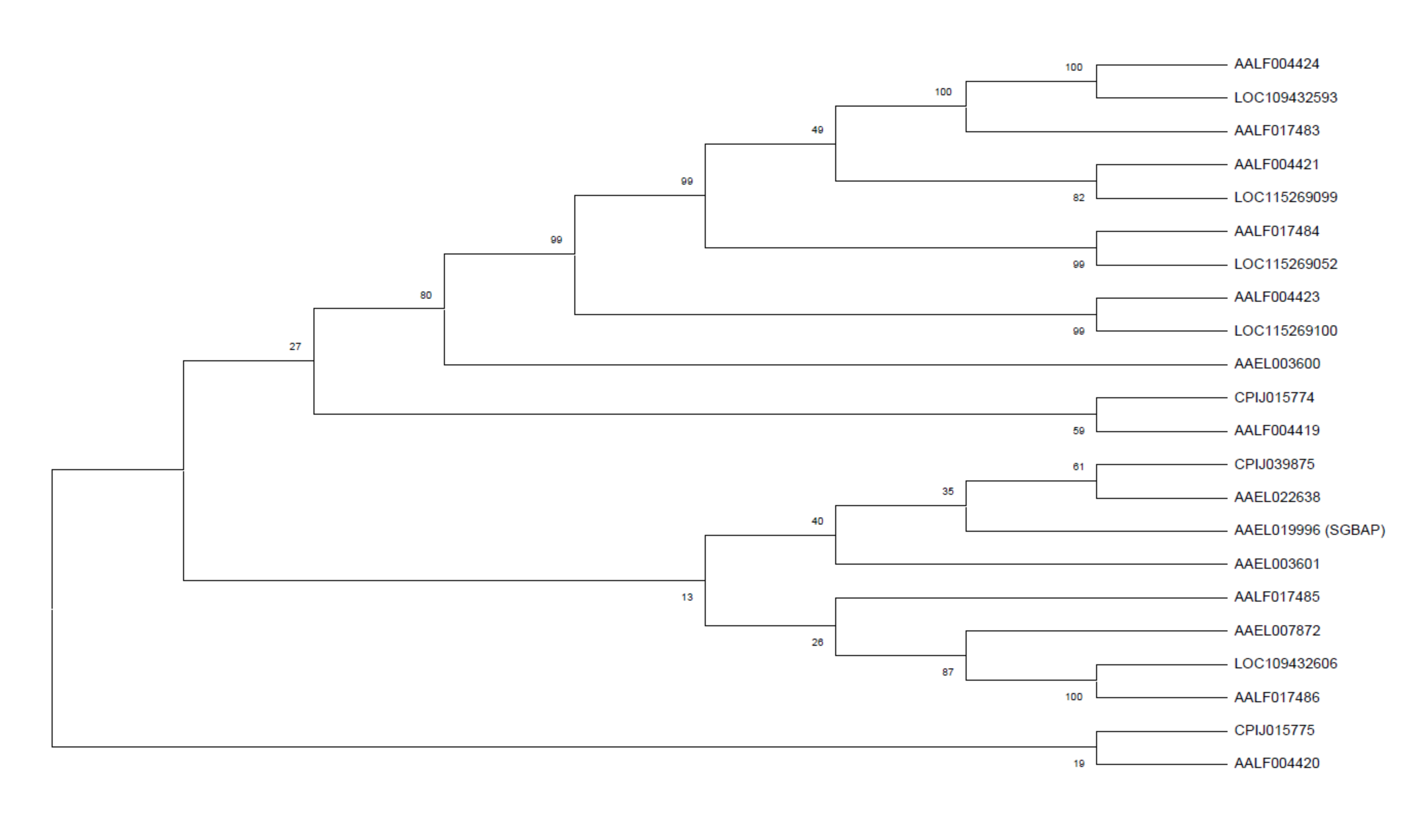

Supplement: Supplementary file 7 — Supplementary Figure S6. [file 41598_2021_3211_MOESM7_ESM.tif]
